# Dietary Effects of Chromium Picolinate and Chromium Nanoparticles in Wistar Rats Fed with a High-Fat, Low-Fiber Diet: The Role of Fat Normalization

**DOI:** 10.3390/nu14235138

**Published:** 2022-12-02

**Authors:** Michał Majewski, Leszek Gromadziński, Ewelina Cholewińska, Katarzyna Ognik, Bartosz Fotschki, Jerzy Juśkiewicz

**Affiliations:** 1 Department of Pharmacology and Toxicology, Faculty of Medicine, University of Warmia and Mazury in Olsztyn, 10-082 Olsztyn, Poland; 2 Department of Cardiology and Internal Medicine, Faculty of Medicine, University of Warmia and Mazury in Olsztyn, 10-082 Olsztyn, Poland; 3 Department of Biochemistry and Toxicology, Faculty of Animal Sciences and Bioeconomy, University of Life Sciences in Lublin, Akademicka 13, 20-950 Lublin, Poland; 4 Institute of Animal Animal Reproduction and Food Research, Polish Academy of Sciences, Tuwima 10, 10-748 Olsztyn, Poland

**Keywords:** 1400 W, acetylcholine, chromium (III), dietary intervention, indomethacin, nanoparticles, NS-398, obesity, thoracic aorta, tranylcypromine

## Abstract

We aimed to evaluate how feeding a high-fat–low-fiber (F) diet to rats and dietary intervention with the implementation of a standard-fat-and-fiber (S) diet affects the response of the cardiovascular system to chromium (III) picolinate (Cr–Pic) and, alternatively, chromium nanoparticles (Cr–NPs). Young male Wistar Han rats (n/group = 12) from either the fatty group (18 weeks on F diet) or the intervention group (9 weeks on F diet + 9 weeks on S diet) received a pharmacologically relevant dose of 0.3 mg Cr/kg body weight in the form of Cr–Pic or Cr–NPs for 9 weeks. Our study on rats confirmed the pro-inflammatory effect of an F diet administered for 18 weeks. In the intervention group, both Cr–Pic and Cr–NPs decreased heart glutathione ratio (GSH+GSSG), enhanced participation of nitric oxide (NO) derived from inducible NO synthase (iNOS) in vascular relaxation to acetylcholine (ACh), increased the vasodilator net effect of cyclooxygenase-2 (COX-2)-derived prostanoids, and increased the production of superoxide anion (O_2_^.−^) in aortic rings. Meanwhile, in the fatty group, there was increased heart superoxide dismutase (SOD), decreased heart catalase (CAT), and reduced sensitivity in pre-incubated aortic rings to endogenous prostacyclin (PGI_2_). The factors that significantly differentiated Cr–NPs from Cr–Pic were (i) decreased blood antioxidant capacity of water-soluble compounds (0.75-fold, *p* = 0.0205), (ii) increased hydrogen peroxide (H_2_O_2_) production (1.59-fold, *p* = 0.0332), and (iii) modified vasodilator response due to PGI_2_ synthesis inhibition (in the intervention group) vs. modified ACh-induced vasodilator response due to (iv) COX inhibition and v) PGI_2_ synthesis inhibition with thromboxane receptor blockage after 18 weeks on F diet (in the fatty group). Our results show that supplementation with Cr–Pic rather than with Cr–NPs is more beneficial in rats who regularly consumed an F diet (e.g., for 18 weeks). On the contrary, in the intervention group (9 weeks on F diet + 9 weeks of dietary fat normalization (the S diet)), Cr–Pic and Cr–NPs could function as pro-oxidant agents, initiating free-radical reactions that led to oxidative stress.

## 1. Introduction

Excessive fat intake, together with obesity, have a negative impact on the cardiovascular system, leading to atherosclerosis with an increased risk of blood clot formation, vascular dysfunction, hypertension, and damage to arteries and many organs such as the heart, brain, kidneys, and eyes [1,2]. Consumption of a high-fat diet, especially over a prolonged period, impairs antioxidant mechanisms, which in turn induces oxidative stress in the body [3,4]. This leads to the overproduction of reactive oxygen and nitrogen species (ROS and RNS), which damage lipids, proteins, and DNA [5].

Vascular relaxation of conduit arteries is regulated by several factors, of which nitric oxide (NO) and arachidonic acid (AA) metabolites are the key mediators [6]. NO-induced vasorelaxation is mediated by soluble guanylate cyclase stimulation, with a further increase in the intracellular cyclic guanosine monophosphate (cGMP) level in the smooth muscle cells of arteries. Prostanoids are derived from AA through the cyclooxygenase (COX) pathway. Among prostanoids, thromboxane A_2_ (TXA_2_) has recently received intensive study due to its pivotal role in cardiovascular disorders [6]. Prostacyclin I_2_ (PGI_2_) is another prostanoid with either vasodilator or vasoconstrictor properties under certain conditions that is widely investigated in vascular disorders [7].

Chromium (Cr) (III) is a popular ingredient in weight-reducing remedies widely sold to the public and is freely available as a preparation for the treatment of type 2 diabetes. However, the European Food Safety Authority (EFSA) concluded that Cr supplementation is not necessary for the proper functioning of the organism [8]. Recent studies have indicated that intake of excessive doses of Cr (III) for a prolonged time period may lead to undesirable processes that modulate the immune response [9,10,11]. Chromium as picolinate (Cr–Pic) is an organic compound of Cr connected to picolinic acid, a tryptophan derivative on the kynurenine pathway [12,13], and is the most popular form available due to its higher absorption (2.8%) compared with inorganic forms (0.9%). Nonetheless, Cr–Pic is poorly absorbed; thus, other chemical compounds are being intensively studied to find a better preparation and to reduce the concentration of Cr. Recently, metal nanoparticles are of interest due to their small size, lack of electric charge, and specific physicochemical properties. On the other hand, these properties make nanoparticles a potential hazard for living organisms, as they easily penetrate biological membranes [5,10,11,12,13,14,15]. There is also a risk that Cr nanoparticles (Cr–NPs) increase oxidative processes in the body and that Cr (with no electric charge) can be transformed into the highly toxic Cr (VI). Metal nanoparticles, such as copper nanoparticles, were recently found to induce a pro-inflammatory response [16,17,18,19], which negatively interfered with the vasodilation of isolated rat thoracic arteries through the modulation of vasoconstrictor prostanoids and their receptors [14].

In our previous studies conducted on rats, supplementation with 0.3 mg of Cr (III) per kg of body weight (BW), particularly in the form of nanoparticles, had a negative impact in vivo, which was reflected in impaired DNA repair and increased DNA oxidation processes in the heart, liver, and brain [1,5]. Cr–NPs reduced the number of white blood cells, which impaired the immune response. Moreover, Cr–Pic and Cr–NPs favor a pro-apoptotic environment with increased content of caspase 3 and 8 in the blood of rats [5].

Bearing all of this in mind, we have postulated that Cr (III), particularly Cr–NPs, may have a negative impact on the heart and vasculature. We aimed to study whether supplementation with Cr–Pic and Cr–NPs (0.3 mg/kg BW) for 9 weeks indeed possesses a harmful effect. The further aim was to evaluate how feeding a high-fat–low-fiber (F) diet to rats for either 9 or 18 weeks and dietary intervention with the implementation of a standard-fat-and-fiber (S) diet affect the redox state of cardiac tissue and the responses of the vascular system to Cr–Pic and Cr–NPs. The antioxidant status of blood plasma and heart was studied together with the participation of arachidonic acid metabolites in the vasodilator response of rat thoracic aorta to acetylcholine ex vivo.

## 2. Materials and Methods

### 2.1. Drugs and Chemicals

The following drugs were used: acetylcholine (ACh) chloride, noradrenaline (NA) hydrochloride, 1400 W, indomethacin, NS-398, SQ-29,548, tranylcypromine (Sigma-Aldrich, Schnelldorf, Germany). Stock solutions were prepared as directed: NA in a mixture of sodium chloride + ascorbic acid (0.9% and 0.01% *w/v*), 1400 W in methanol, SQ-29,548 and indomethacin in ethanol, and TCP and NS-398 in DMSO. Other drugs were prepared in distilled water. The stock solutions (10 mM) were kept at −20 °C. All dilutions were made in a Krebs–Henseleit buffer (KH buffer: mM; NaCl 115, CaCl_2_ 2.5, KCl 4.6, KH_2_PO_4_ 1.2, MgSO_4_ 1.2, NaHCO_3_ 25, and glucose 11.1 at pH 7.4) on the day of the experiment. The maximal solvent concentration in organ baths was less than 0.01% (*v/v*).

Chromium nanoparticles/nanopowder of 99.9% trace metals basis purity, particle size (APS) 60–80 nm, specific surface area (SSA) 6–8 m^2^/g, spherical morphology, bulk density ~0.15 g/cm^3^, and true density 8.9 g/cm^3^ were purchased from SkySprings Nanopowders (Houston, TX, USA). Chromium picolinate (Cr–Pic) and chromium nanoparticles (Cr–NPs) were added to the diet as an emulsion together with dietary rapeseed oil.

### 2.2. Animal Protocol and Dietary Treatment

**Figure 1:**
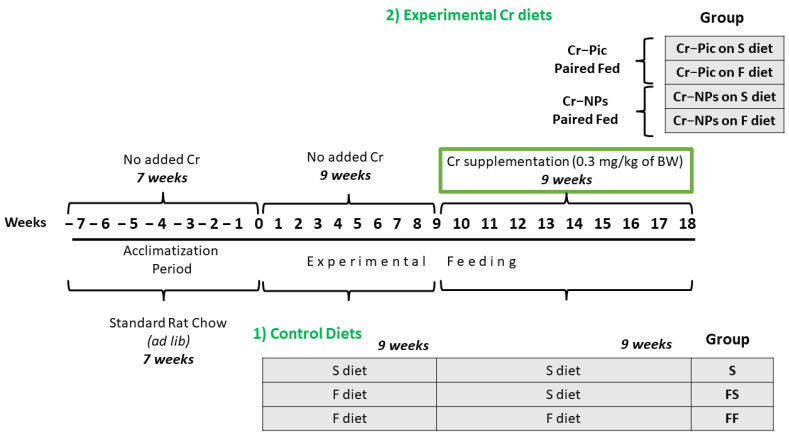
Animal feeding groups and experimental timeline. 0.3 mg Cr/kg of body weight as chromium picolinate (Cr-Pic) and chromium nanoparticles (Cr-NPs) were added to the diet as an emulsion together with dietary rapeseed oil for 9 weeks of supplementation after the initial 9 week period of experimental feed without Cr supplementation. Rats at 7 weeks of age were fed with two types of diet: a standard-fat-and-fiber (S) diet and a high-fat–low-fiber (F) diet for either 9 weeks or 18 weeks (9 + 9 weeks). The following groups of rats were studied: Control S: the S diet plus the S diet; Control FS: the F diet and the S diet; Cr–Pic FS: the F diet and the Cr–Pic S diet; Cr–NPs FS: the F diet and the Cr–NPs S diet; Control FF: the F diet and the F diet; Cr–Pic FF: the F diet and the Cr–Pic F diet; Cr–NPs FF: the F diet and the Cr–NPs F diet.

Male Wistar Han rats from the Institute of Animal Reproduction and Food Research PAS, Olsztyn, Poland were randomly divided at 7 weeks of age into 7 groups of 12 animals each, see Figure 1.

Experimental feeding consisted of two 9-week periods, i.e., initial and experimental, wherein 0.3 mg Cr/kg of body weight as chromium picolinate (Cr–Pic) and chromium nanoparticles (Cr-NPs) were added for 9 weeks of supplementation after the initial 9 week period of experimental feed without Cr supplementation. Two types of diet were applied (see Table 1): a standard-fat-and-fiber (S) diet and a high-fat–low-fiber (F) diet. The latter diet was a modification of the S diet, with 17% lard replacing maize starch and with only 3% cellulose content instead of 8%. Rats were fed with the S diet or F diet for either 9 weeks or 18 weeks.

This study was conducted on two main groups:

(A) Control, which was further subdivided into:
  1. Control S (the S diet and the S diet)
  2. Control FS (the F diet and the S diet)
  3. Control FF (the F diet and the F diet)
(B) Supplemented, which was further subdivided into:
  1. Cr–Pic FS (the FS diet supplemented with Cr–Pic)
  2. Cr–NPs FS (the FS diet supplemented with Cr–NPs)
  3. Cr–Pic FF (the FF diet supplemented with Cr–Pic)
  4. Cr–NPs FF (the FF diet supplemented with Cr–NPs)

The concentration of Cr in the S diet was 1.26 mg/kg, which, in terms of Cr–Pic and Cr–NPs, corresponded to concentrations of 4.01 and 4.05 mg/kg as measured with ASA (AA-7000, Shimadzu). Each rat received chromium in the amount of 0.3 mg/kg of BW, which was calculated daily from the amount of daily food intake and the body weight. For safety reasons, Cr preparations were prepared in the form of emulsion in rapeseed oil and added into the diet [10].

Rats were kept in separate metabolic cages under the following conditions: temperature of 21–22 °C, relative humidity of 60% ± 10%, and a ventilation rate of 15 air changes during one hour. Rats had free access to a fresh diet and water on a daily basis.

### 2.3. Experimental Procedures

Intraperitoneal injection of ketamine/xylazine (100/10 mg/kg BW) was used for anesthesia. Rats were killed by decapitation and exsanguinated. Blood samples were kept in vials containing heparin + EDTA as an anticoagulant and centrifuged at 3000× *g* for 10 min to separate the blood plasma. The hearts were carefully dissected, weighed, and immediately placed in liquid nitrogen (−196 °C) for 30 min and then stored at low temperature (−80 °C) for further analyses. The thoracic aorta was dissected with care, cleaned of adherent tissue, cut into 6–8 rings, and kept on ice.

For the extraction and quantification of proteins, rat tissue was homogenized with the RIPA buffer (Santa Cruz Biotechnology, Heidelberg, Germany), with absorbance set at 595 nm. Bovine serum albumin was used as a control [20].

### 2.4. Blood Pressure

Heart rate (bpm) and mean arterial pressure (mmHg) were measured one day before the blood collection with the noninvasive tail-cuff method (LE5001, Panlab, Harvard Apparatus, Barcelona, Spain) [21].

### 2.5. The Antioxidant Capacity of Blood Plasma

The antioxidant capacity of water (ACW)- and lipid (ACL)-soluble compounds of the blood plasma (in µg/mL) was determined with Photochem (Analytik Jena AG, Jena, Germany). This photo-chemiluminescence detection method generates free radicals that are removed with the antioxidants presented in blood plasma, and the remaining radicals are quantified. The calibration curve was prepared with ascorbic acid and Trolox as standards for ACW and ACL [21].

### 2.6. Markers of Antioxidant Status in the Heart

Heart malondialdehyde (MDA, nmol/g), the total sum of reduced and oxidized glutathione (GSH+GSSG, nmol/g), superoxide dismutase (SOD, U/g), and catalase (CAT, U/g) were analyzed according to a previously described method [2,14]. The MDA reacts with thiobarbituric acid (TBA), which gives an MDA–TBA adduct that was quantified with a fluorometric assay kit (ab118970) at Ex/Em = 532/553 nm. GSH+GSSG and SOD activities were determined with Ransel and Ransod colorimetric diagnostic kits from Randox (Warsaw, Poland), following the manufacturer’s instructions. CAT activity was determined with an Oxis International, Inc., (Portland, OR, USA) diagnostic kit following the manufacturer’s instructions.

### 2.7. Vascular Reactivity Studies

Aortic rings from the thoracic segment of the aorta (4–5 mm length) were mounted in stagnant 5 mL chambers (Graz Tissue Bath System, Barcelona, Spain) and aerated with carbogen gas for 60 min. Two parallel stainless steel wires were implemented through the lumen of the aortic rings: one fixed to the bath wall and the other connected to a force transducer (FT20, TAM-A, Hugo Sachs Elektronik, March, Germany). Pre-load tension of 1 cN was applied to the tissue (measured with LabChart, ADInstruments, New South Wales, Australia). The functional integrity of aortic rings was checked with high KCl (75 mM) and ACh (10 µM). Next, aortic rings were pre-incubated for 30 min with either the inducible nitric oxide synthase (iNOS) inhibitor (1400 W at 1 µM), the non-selective COX inhibitor (indomethacin at 10 µM), the selective cyclooxygenase-2 (COX-2) inhibitor (NS-398 at 10 µM), the thromboxane-A_2_ receptor (TP) antagonist (SQ-29,548 at 1 µM), the PGI_2_ synthesis inhibitor (tranylcypromine, TCP at 10 µM), or TCP (10 µM) plus SQ-29,548 (1 µM) and contracted with noradrenaline (0.1 µM). Afterwards, the cumulative concentrations of ACh (0.1 nM–10 µM) were added into the incubation chambers, and this was done once only. In another set of experiments, the cumulative concentrations of NA (0.1 nM–10 µM) and SNP (0.1 nM–10 µM) were constructed.

### 2.8. Biochemical Studies of Aortic Rings

#### 2.8.1. TXA_2_ and PGI_2_ Production

First, we stabilized the aortic rings from each group of rats in KHS at +37 °C for 30 min at pH = 7.4. Next, aortic rings were washed twice with 200 μL of KHS for 10 min. Further, we exposed aortic rings to noradrenaline (0.1 μM, 2 min) and to increasing concentrations of ACh (0.1 nM–10 μM, at 1-min intervals). Production of TXA_2_ and PGI_2_ were monitored by measuring the release of their stable metabolites, TxB_2_ and 6-keto-PGF1α, with the appropriate enzyme immunoassay kit (Cayman Chemical, Ann Arbor, MI, USA). Results were expressed as pg prostanoid/mg of protein [6].

#### 2.8.2. Detection of Superoxide Anion

This was measured using lucigenin chemiluminescence, as previously described [20]. Aortic rings without endothelium were equilibrated in HEPES buffer at 37 °C for 30 min and moved to test tubes containing lucigenin (5 µmol/L) + 1 mL HEPES buffer (pH = 7.4, at 37 °C). Tiron (10 mmol/L), a superoxide anion (O_2_^.−^) scavenger, was added for chemiluminescence detection. For background emission, blank samples without aortic rings were prepared. Results were expressed as chemiluminescence units CU/minute/mg tissue.

#### 2.8.3. Detection of Hydrogen Peroxide

This was measured with a fluorescence assay kit (Cayman Chemicals, Ann Arbor, MI, USA) at 530/590 nm excitation/emission wavelengths following the manufacturer’s instructions. To ensure the specificity of the method, some of the rings were subjected to CAT, a hydrogen peroxide (H_2_O_2_) scavenger. Results were expressed as nmol/µg protein.

### 2.9. Data Analysis and Statistics

Results are given as means ± SD or means ± SEM (for vascular reactivity studies) and were prepared in GraphPad Prism 8.4. Vascular relaxation to ACh was calculated as a percentage of the response to NA and analyzed with a nonlinear regression model. This determined the area under the curve (AUC), the maximal response (Emax, %), and the potency (the negative logarithm of the concentration causing a half-maximum effect, pEC_50_). The obtained data were checked with Grubbs’ test and were analyzed for Gaussian distribution of residuals and homoscedasticity of variance. The group comparison was performed by two-way ANOVA with an appropriate post hoc test. Differences were considered significant when *p* ≤ 0.05.

## 3. Results

### 3.1. General Characteristics

Blood pressure did not differ between the studied groups (data not presented).

### 3.2. The Antioxidant Capacity of Blood Plasma

#### 3.2.1. Blood Plasma ACW

**Figure 2:**
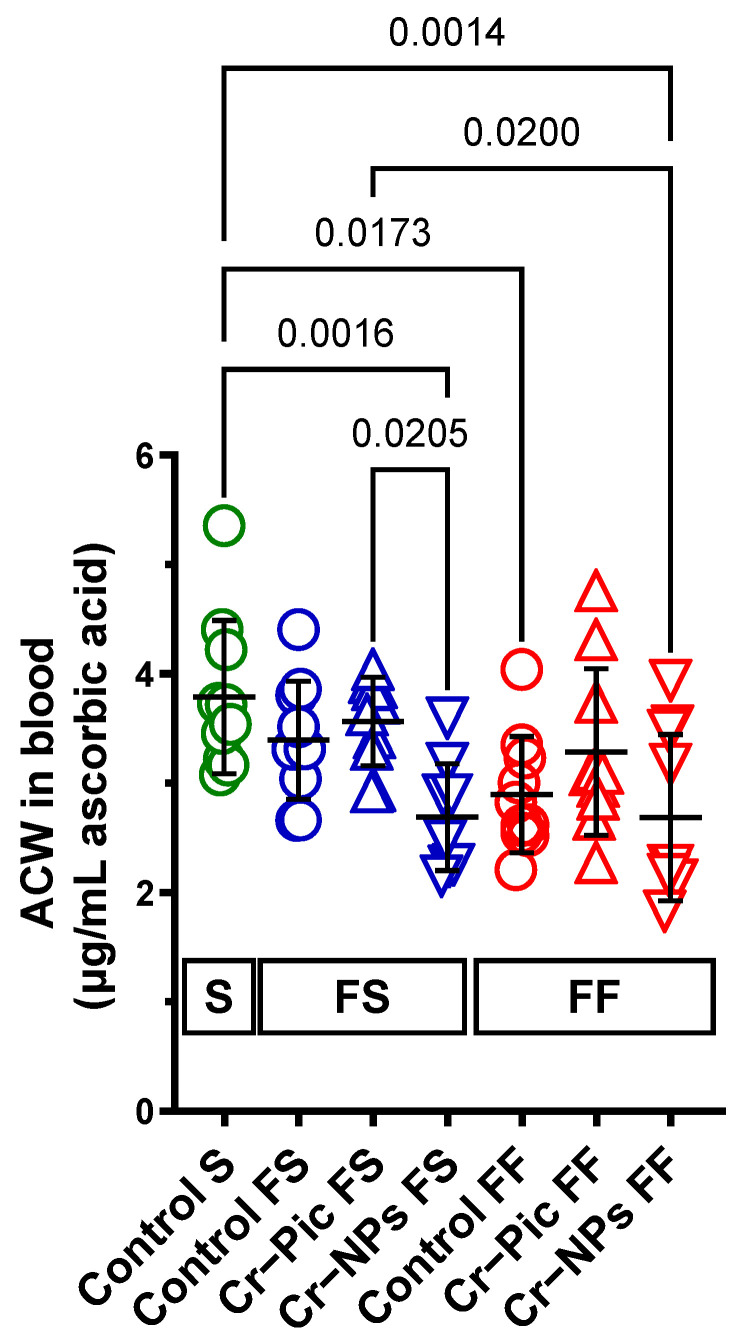
Blood plasma ACW of either chromium (Cr)-supplemented (9 weeks of supplementation) or non-supplemented (Control S, Control FS, and Control FF) rats. 0.3 mg of Cr per kg of body weight as chromium picolinate (Cr–Pic) or chromium nanoparticles (Cr–NPs) were added after the initial 9 weeks of experimental feed without Cr supplementation. Two types of diet were applied: a standard-fat-and-fiber (S) diet and a high-fat–low-fiber (F) diet. Rats at 7 weeks of age were fed with an S diet or an F diet for either 9 weeks or 9 + 9 weeks. The following groups of rats were studied: Control S, the S diet plus the S diet; Control FS, the F diet and the S diet; Cr–Pic FS, the F diet and the Cr–Pic S diet; Cr–NPs FS, the F diet and the Cr–NPs S diet; Control FF, the F diet and the F diet; Cr–Pic FF, the F diet and the Cr–Pic F diet; Cr–NPs FF, the F diet and the Cr–NPs F diet. Values are means ± SD, *n* = 10, *p* ≤ 0.05 (two-way ANOVA/Tukey’s multiple comparisons test). ACW is a marker that distinguishes Cr–NPs from Cr–Pic in rats fed with the F diet for 9 weeks. Abbreviations: ACW, antioxidant capacity of water-soluble compounds.

When the F diet was administered for 9 weeks, ACW did not change in the control FS group (S group vs. FS control, *p* = 0.7225). However, Cr–NPs decreased the ACW compared to the S group (0.71-fold, *p* = 0.0016), see Figure 2. A significant decrease was observed in the Cr–NPs group vs. the Cr–Pic group (0.75-fold, *p* = 0.0205).

When the F diet was administered for 18 weeks, ACW decreased in the control FF group (S group vs. FF control, 0.76-fold, *p* = 0.0173). Cr–NPs, but not Cr–Pic, decreased the ACW compared to the S group (0.71-fold, *p* = 0.0014).

We did not observe any statistically significant change due to the dietary intervention between 9 weeks and 18 weeks of F diet for Cr-supplemented or non-supplemented (control) rats.

#### 3.2.2. Blood Plasma ACL

ACL did not differ between the studied groups (5.339 ± 1.446, *p* ≥ 0.8818, data not presented).

### 3.3. Markers of Antioxidant Status in the Heart

#### 3.3.1. Heart Malondialdehyde

**Figure 3:**
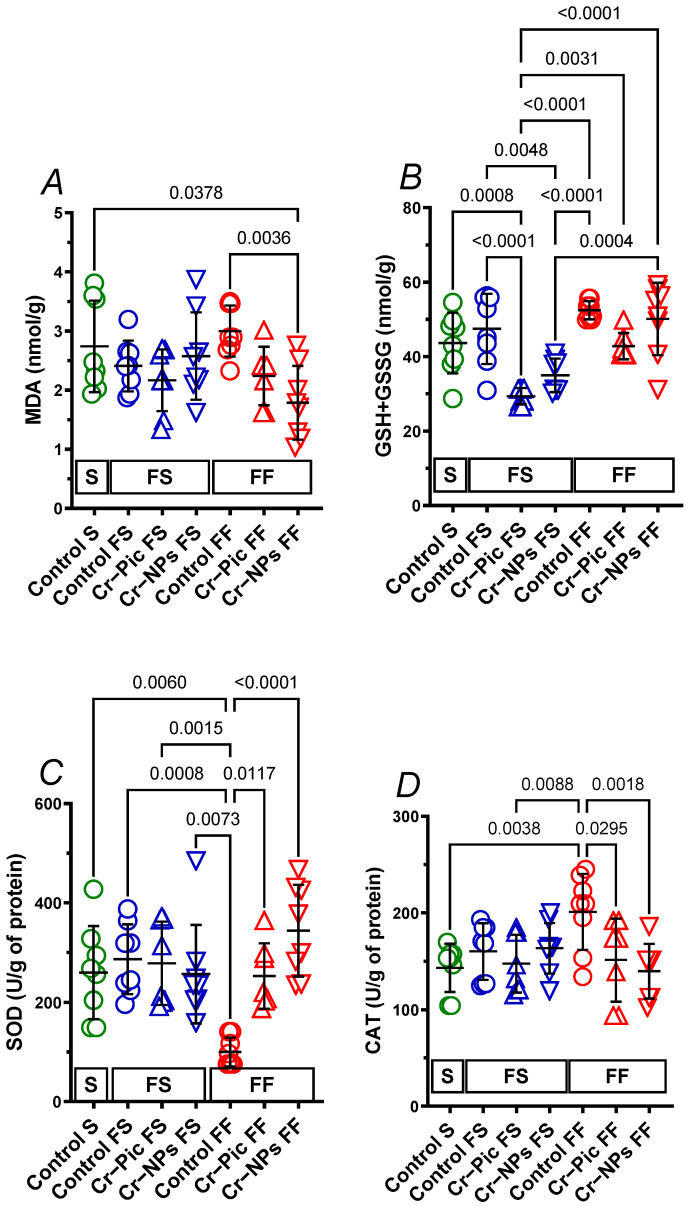
Heart MDA (**A**), GSH+GSSG (**B**), SOD (**C**) and CAT (**D**) of either chromium (Cr)-supplemented (9 weeks of supplementation) or nonsupplemented (Control S, Control FS, and Control FF) rats. 0.3 mg Cr/kg of body weight as chromium picolinate (Cr–Pic) or chromium nanoparticles (Cr–NPs) were added after the initial 9 weeks of experimental feed without Cr supplementation. Two types of diet were applied: a standard-fat-and-fiber (S) diet and a high-fat–low-fiber (F) diet. Rats at 7 weeks of age were fed with an S diet or an F diet for either 9 weeks or 9 + 9 weeks. The following groups of rats were studied: Control S, the S diet plus the S diet; Control FS, the F diet and the S diet; Cr–Pic FS, the F diet and the Cr–Pic S diet; Cr–NPs FS, the F diet and the Cr–NPs S diet; Control FF, the F diet and the F diet; Cr–Pic FF, the F diet and the Cr–Pic F diet; Cr–NPs FF, the F diet and the Cr–NPs F diet. Values are means ± SD, n = 8, *p* ≤ 0.05 (two-way ANOVA/Tukey’s multiple comparisons test). In rats fed with the F diet for 9 weeks, Cr–NPs and Cr–Pic decreased the GSH+GSSG. In rats fed with F diet for 18 weeks Cr–NPs decreased both the MDA and CAT and increased SOD; meanwhile, Cr–Pic decreased CAT and increased SOD. No significant difference between Cr–NPs and Cr–Pic was observed in MDA, GSH+GSSG, SOD, and CAT. Abbreviations: CAT, catalase; GSH+GSSG, total glutathione; MDA, malondialdehyde; SOD, superoxide dismutase.

When the F diet was administered for 9 weeks, MDA did not change in the control FS group (S group vs. FS control, *p* = 0.9191). Neither Cr–NPs nor Cr–Pic changed the MDA, see Figure 3A.

When the F diet was administered for 18 weeks, MDA did not change in the control FF group (S group vs. FF control, *p* = 0.9758). However, it was Cr–NPs, not Cr–Pic, that decreased MDA compared to the control FF group (0.60-fold, *p* = 0.0036) and the S group (0.65-fold, *p* = 0.0378).

We did not observe any significant change due to dietary intervention between 9 weeks and 18 weeks of F diet in Cr-supplemented or non-supplemented (control) rats.

#### 3.3.2. Heart GSH+GSSG

When the F diet was administered for 9 weeks, GSH+GSSG did not change in the control FS group (S group vs. FS control, *p* = 0.8875). Cr–Pic decreased GSH+GSSG compared to the control FS group (0.62-fold, *p* < 0.0001) and the S group (0.67-fold, *p* = 0.0008). Meanwhile, Cr–NPs decreased GSH+GSSG compared to the control FS group (0.74-fold, *p* = 0.0048), but not the S group (0.80-fold, *p* = 0.1074), see Figure 3B.

When the F diet was administered for 18 weeks, GSH+GSSG tended to increase in the control FF group (S group vs. FF control, 1.2-fold, *p* = 0.0973). Cr–Pic tended to decrease GSH+GSSG compared to the control FF group (0.82-fold, *p* = 0.0704).

However, we did observe a significant increase in GSH + GSSG during 18 weeks of F diet fed Cr–Pic and Cr–NPs rats by 1.46-fold, *p* = 0.0031 and 1.43-fold, *p* = 0.0004, respectively. This was not observed in non-supplemented controls (*p* = 0.6796), see Figure 3B.

#### 3.3.3. Heart SOD

When the F diet was administered for 9 weeks, SOD did not change in the control FS group (S group vs. FS control, *p* = 0.9939). Neither Cr–NPs nor Cr–Pic changed the SOD, see Figure 3C.

When the F diet was administered for 18 weeks, SOD decreased in the control FF group (S group vs. FF control, 0.39-fold, *p* = 0.0060), and both Cr–NPs and Cr–Pic increased SOD compared to the control FF group (3.44-fold, *p* < 0.0001 and 2.52-fold, *p* = 0.0117, respectively).

We did not observe any significant change due to the dietary intervention between 9 weeks and 18 weeks of F diet in Cr-supplemented rats. However, a significant decrease was observed in non-supplemented control FF rats (0.35-fold, *p* = 0.0008).

#### 3.3.4. Heart CAT

When the F diet was administered for 9 weeks, CAT did not change in the control FS group (S group vs. FS control, *p* = 0.8968). Neither Cr–NPs nor Cr–Pic changed the CAT, see Figure 3D.

When the F diet was administered for 18 weeks, CAT increased in the control FF group (S group vs. FF control, 1.40-fold, *p* = 0.0038), and both Cr–NPs and Cr–Pic decreased CAT compared to the control FF group (0.70-fold, *p* = 0.0018 and 0.75-fold, *p* = 0.0295, respectively).

We did not observe any significant change due to the dietary intervention between 9 weeks and 18 weeks of F diet in Cr-supplemented or non-supplemented (control) rats.

### 3.4. Vascular Studies

#### 3.4.1. Vascular Reactivity Studies

Vasoconstrictor response to high KCl (75 mM) and NA (0.1 nM–10 μM) did not differ between the studied groups (data not presented).

**Figure 4:**
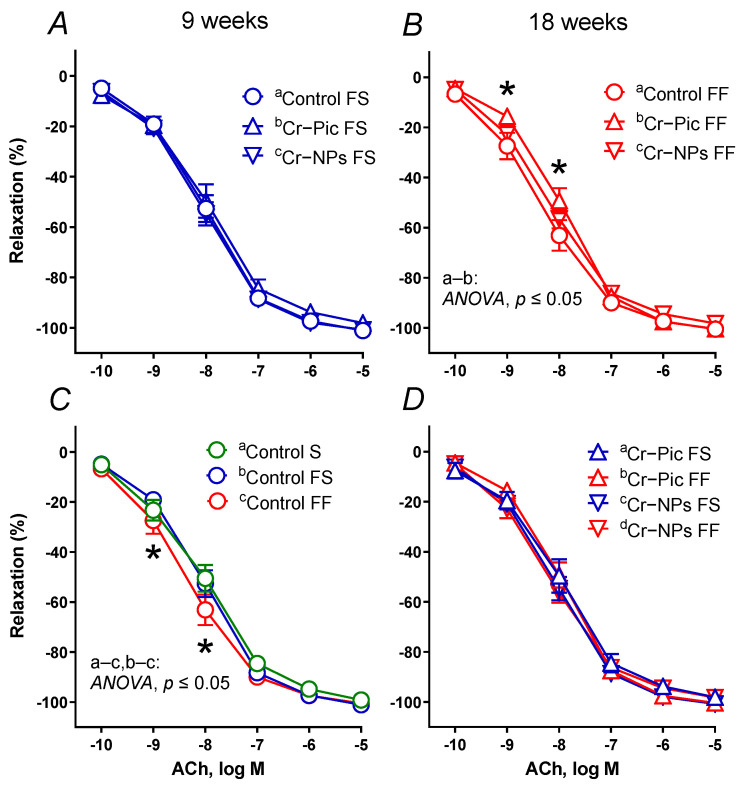
The vasodilator response to acetylcholine (ACh) in aortic rings from either chromium (Cr) supplemented (9 weeks of supplementation) or non-supplemented (Control S, Control FS, and Control FF) rats. 0.3 mg Cr/kg of body weight as chromium picolinate (Cr–Pic) or chromium nanoparticles (Cr–NPs) were added after the initial 9 weeks of experimental feed without Cr supplementation. Two types of diet were applied: a standard-fat-and-fiber (S) diet and a high-fat–low-fiber (F) diet. Rats at 7 weeks of age were fed with an S diet or an F diet for either 9 weeks or 9 + 9 weeks. The following groups of rats were studied: Control S, the S diet plus the S diet; Control FS, the F diet and the S diet; Cr–Pic FS, the F diet and the Cr–Pic S diet; Cr–NPs FS, the F diet and the Cr–NPs S diet; Control FF, the F diet and the F diet; Cr–Pic FF, the F diet and the Cr–Pic F diet; Cr–NPs FF, the F diet and the Cr–NPs F diet. FS group (**A**), FF group (**B**), Control groups (**C**), FS vs. FF group (**D**). Results (means ± SEM) are expressed as a percentage of the inhibition of the contraction induced by noradrenaline (0.1 μM), *n* = 8, * *p* ≤ 0.05 (two-way ANOVA/Šídák’s). F diet given for 18 weeks decreased vasodilation to ACh compared to either S diet (18 weeks) or F diet (9 weeks), and it was Cr–Pic that modified vasodilation.

Vasodilator response to SNP did not differ between the studied groups (data not presented), as opposed to the ACh-induced response, see Figure 4A–D.

When the F diet was administered for 9 weeks, the vasodilator response to ACh did not change in the control FS group (Figure 4C). Neither Cr–NPs nor Cr–Pic changed the vasodilation (Figure 4A).

When the F diet was administered for 18 weeks, the vasodilator response to ACh shifted to the left in the control FF group. However, it was Cr–Pic, not Cr–NPs that shifted the vasodilation back to the right (Figure 4B). We did not observe any significant change due to the dietary (F) intervention (9 weeks vs. 18 weeks) in Cr–Pic- and Cr–NPs-supplemented rats (Figure 4D). However, a significant change was observed between non-supplemented controls (control FS vs. control FF, Figure 4C).

**Figure 5:**
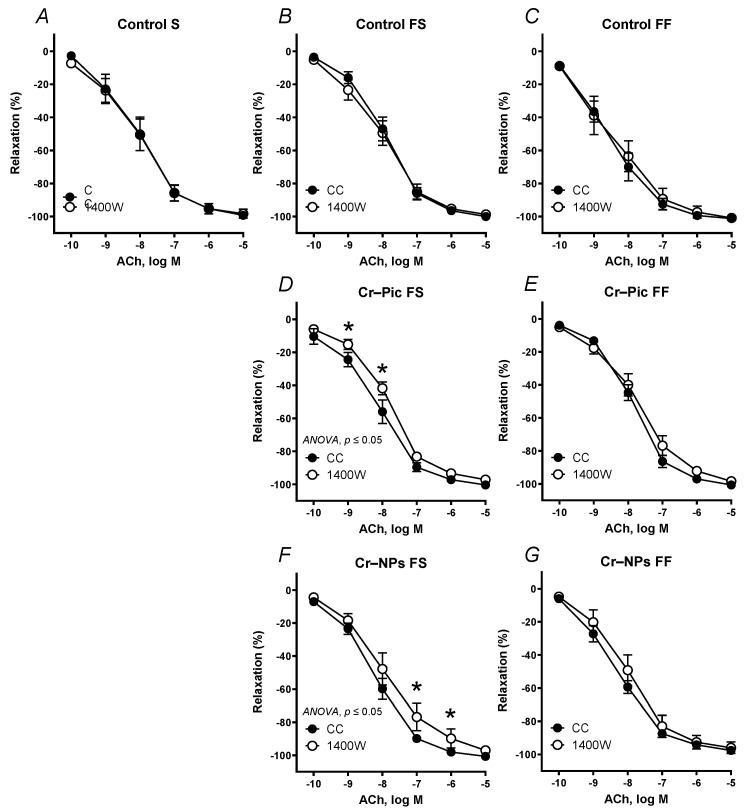
Effects of 1400 W (1 µM) on the concentration–response curves to acetylcholine (ACh) in aortic rings from the following groups of rats: control S (**A**), control FS (**B**), control FF (**C**), Cr–Pic FS (**D**), Cr–Pic FF (**E**), Cr–NPs FS (**F**), and Cr–NPs FF (**G**). 0.3 mg Cr/kg of body weight as chromium picolinate (Cr–Pic) and chromium nanoparticles (Cr–NPs) were added for 9 weeks of supplementation after the initial 9-week period of experimental feed without Cr supplementation. Two types of diet were applied: a standard-fat-and-fiber (S) diet and a high-fat–low-fiber (F) diet. Rats at 7 weeks of age were fed with an S diet or an F diet for either 9 weeks or 9 + 9 weeks. The following groups of rats were studied: Control S, the S diet plus the S diet; Control FS, the F diet and the S diet; Cr–Pic FS, the F diet and the Cr–Pic S diet; Cr–NPs FS, the F diet and the Cr–NPs S diet; Control FF, the F diet and the F diet; Cr–Pic FF, the F diet and the Cr–Pic F diet; Cr–NPs FF, the F diet and the Cr–NPs F diet. Results (means ± SEM) are expressed as percentage of inhibition of contraction induced by noradrenaline (0.1 µM). *n* = 8, * *p* ≤ 0.05 (two-way ANOVA/Šídák’s) compared with control conditions. In arteries from rats fed with the F diet for 9 weeks, both Cr–Pic and Cr–NPs enhanced participation of NO derived from iNOS in vascular relaxation to ACh.

Pre-incubation of the aortic rings with the inducible nitric oxide synthase (iNOS) inhibitor 1400 W did not change the vasodilator response to ACh in arteries compared to the control groups of rats (control S, control FS, and control FF, Figure 5A–C).

When the F diet was administered for 9 weeks, pre-incubation with 1400 W shifted the ACh-induced response to the right in Cr–Pic and Cr–NPs groups (Figure 5D,F).

When the F diet was administered for 18 weeks, pre-incubation with 1400 W did not change vasodilation in all studied groups (Figure 5E,G).

**Figure 6:**
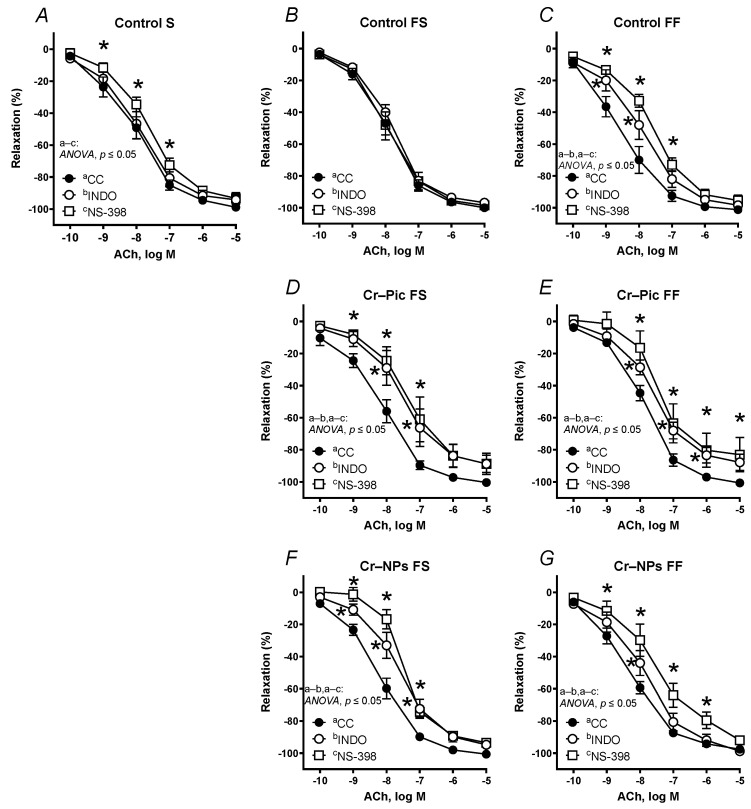
Effects of indomethacin (10 µM) and NS-398 (10 µM) on the concentration–response curves to acetylcholine (ACh) in aortic rings from the following groups of rats: control S (**A**), control FS (**B**), control FF (**C**), Cr–Pic FS (**D**), Cr–Pic FF (**E**), Cr–NPs FS (**F**), and Cr–NPs FF (**G**). 0.3 mg Cr/kg of body weight as chromium picolinate (Cr–Pic) and chromium nanoparticles (Cr–NPs) were added for 9 weeks of supplementation after the initial 9-week period of experimental feed without Cr supplementation. Two types of diet were applied: a standard-fat-and-fiber (S) diet and a high-fat–low-fiber (F) diet. Rats at 7 weeks of age were fed with an S diet or an F diet for either 9 weeks or 9 + 9 weeks. The following groups of rats were studied: Control S, the S diet plus the S diet; Control FS, the F diet and the S diet; Cr–Pic FS, the F diet and the Cr–Pic S diet; Cr–NPs FS, the F diet and the Cr–NPs S diet; Control FF, the F diet and the F diet; Cr–Pic FF, the F diet and the Cr–Pic F diet; Cr–NPs FF, the F diet and the Cr–NPs F diet. Results (means ± SEM) are expressed as percentage of inhibition of contraction induced by noradrenaline (0.1 µM). n = 8, * *p* ≤ 0.05 (two-way ANOVA/Šídák’s) compared with control conditions. In arteries from rats fed with the F diet for 9 weeks, both Cr–Pic and Cr–NPs attenuated vasodilation.

Pre-incubation of the aortic rings with the specific COX-2 inhibitor NS-398 shifted the vasodilator response to the right in ACh in arteries from the control S rats. A non-selective COX inhibitor indomethacin did not modify that response (Figure 6A).

When the F diet was administered for 9 weeks, pre-incubation with NS-398 and indomethacin shifted the ACh-induced response to the right in Cr–Pic- and Cr–NPs-supplemented rats (Figure 6D,F), but not in the control group (Figure 6B). Moreover, the maximal response (Emax) decreased in arteries from the Cr–Pic group (Figure 6D).

When the F diet was administered for 18 weeks, pre-incubation with NS-398 and indomethacin shifted vasodilation to the right in the control group (Figure 6C) and in the two Cr groups (Cr–Pic and Cr–NPs, Figure 6E,G). Moreover, the maximal response (Emax) decreased in arteries from Cr–Pic-supplemented rats only (Figure 6E).

**Figure 7:**
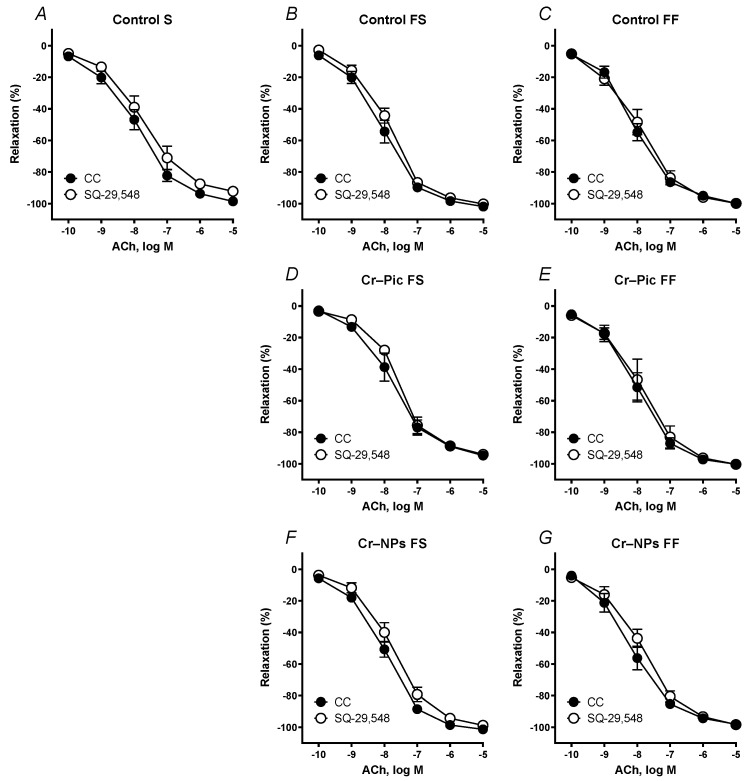
Effects of SQ-29,548 (1 µM) on the concentration–response curves to acetylcholine (ACh) in aortic rings from the following groups of rats: control S (**A**), control FS (**B**), control FF (**C**), Cr–Pic FS (**D**), Cr–Pic FF (**E**), Cr–NPs FS (**F**), and Cr–NPs FF (**G**). 0.3 mg Cr/kg of body weight as chromium picolinate (Cr–Pic) and chromium nanoparticles (Cr–NPs) were added for 9 weeks of supplementation after the initial 9-week period of experimental feed without Cr supplementation. Two types of diet were applied: a standard-fat-and-fiber (S) diet and a high-fat–low-fiber (F) diet. Rats at 7 weeks of age were fed with an S diet or an F diet for either 9 weeks or 9 + 9 weeks. The following groups of rats were studied: Control S, the S diet plus the S diet; Control FS, the F diet and the S diet; Cr–Pic FS, the F diet and the Cr–Pic S diet; Cr–NPs FS, the F diet and the Cr–NPs S diet; Control FF, the F diet and the F diet; Cr–Pic FF, the F diet and the Cr–Pic F diet; Cr–NPs FF, the F diet and the Cr–NPs F diet. Results (means ± SEM) are expressed as percentage of inhibition of contraction induced by noradrenaline (0.1 µM). n = 8, *p* ≤ 0.05 (two-way ANOVA/Šídák’s) compared with control conditions. No significant difference was observed between the analyzed groups.

Pre-incubation of the aortic rings with the TXA_2_ (TP) receptor antagonist SQ-29,548 (1 mmol/L, 30 min) did not modify the response to ACh in arteries from all studied groups of rats (Figure 7A–G).

**Figure 8:**
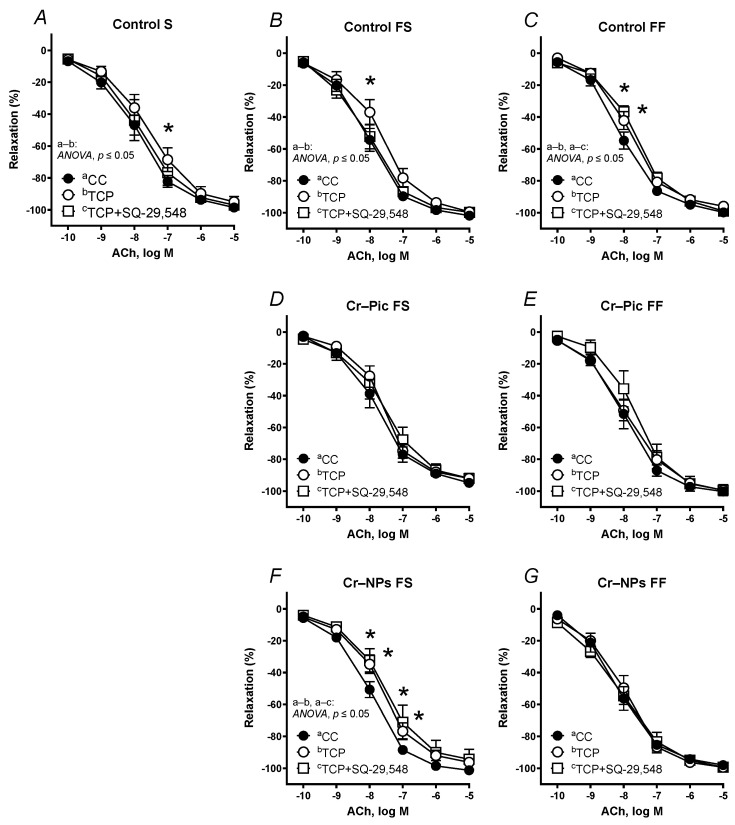
Effects of TCP (10 µM) and TCP (10 µM) plus SQ-29,548 (1 µM) on the concentration–response curves to acetylcholine (ACh) in aortic rings from the following groups of rats: control S (**A**), control FS (**B**), control FF (**C**), Cr–Pic FS (**D**), Cr–Pic FF (**E**), Cr–NPs FS (**F**), and Cr–NPs FF (**G**). 0.3 mg Cr/kg of body weight as chromium picolinate (Cr–Pic) and chromium nanoparticles (Cr–NPs) were added for 9 weeks of supplementation after the initial 9-week period of experimental feed without Cr supplementation. Two types of diet were applied: a standard-fat-and-fiber (S) diet and a high-fat–low-fiber (F) diet. Rats at 7 weeks of age were fed with an S diet or an F diet for either 9 weeks or 9 + 9 weeks. The following groups of rats were studied: Control S, the S diet plus the S diet; Control FS, the F diet and the S diet; Cr–Pic FS, the F diet and the Cr–Pic S diet; Cr–NPs FS, the F diet and the Cr–NPs S diet; Control FF, the F diet and the F diet; Cr–Pic FF, the F diet and the Cr–Pic F diet; Cr–NPs FF, the F diet and the Cr–NPs F diet. Results (means ± SEM) are expressed as a percentage of inhibition of contraction induced by noradrenaline (0.1 µM). *n* = 8, * *p* ≤ 0.05 (two-way ANOVA/Šídák’s) compared with control conditions. In arteries from rats fed with the F diet for 9 weeks, Cr–NPs decreased the sensitivity to ACh in arteries pre-incubated with TCP and TCP+SQ-29,548.

Pre-incubation of the aortic rings with the specific PGI_2_ synthesis inhibitor TCP shifted the vasodilator response to ACh in arteries to the right in all three control groups: control S, control FS and, control FF (Figure 8A–C).

When the F diet was administered for 9 weeks, pre-incubation with TCP shifted the ACh-induced response to the right in the arteries from Cr–NPs rats, but not in the Cr–Pic group (Figure 8B,D,F).

When the F diet was administered for 18 weeks, pre-incubation with TCP did not modify the vasodilation in Cr–NPs and Cr–Pic arteries (Figure 8C,E,G).

Pre-incubation with TCP plus SQ-29,548 normalized the ACh-induced response to the control level in arteries from the control S and control FS groups but not in the control FF group (Figure 8A–C).

When the F diet was administered for 9 weeks, pre-incubation with TCP plus SQ-29,548 did not modify the vasodilator response to ACh in arteries from Cr–Pic rats (Figure 8D), while it was not normalized in arteries from Cr–NPs rats (Figure 8F).

When the F diet was administered for 18 weeks, pre-incubation with TCP plus SQ-29,548 did not modify the vasodilation in Cr–NPs and Cr–Pic arteries (Figure 8E,G).

For AUC, Emax (%), and pEC_50_, see Table 2.

#### 3.4.2. TXA_2_ and PGI_2_ Production

Neither Cr–Pic nor Cr–NPs modified the production of TXA_2_ and PGI_2_ over 9 and 18 weeks of F diet administration (data not presented).

#### 3.4.3. Detection of Superoxide Anion

**Figure 9:**
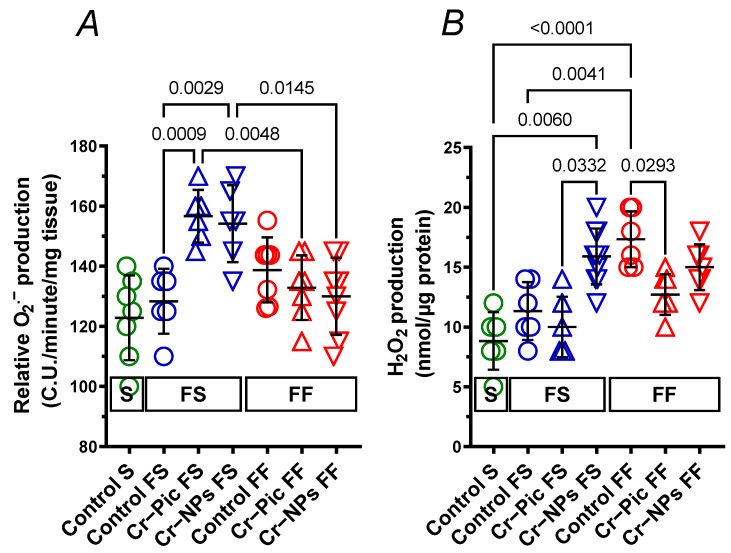
Production of superoxide anion (**A**) and hydrogen peroxide (**B**) in aortic rings from either chromium (Cr) supplemented (9 weeks of supplementation) or non-supplemented (Control) rats. 0.3 mg Cr/kg of body weight as chromium picolinate (Cr–Pic) and chromium nanoparticles (Cr–NPs) were added for 9 weeks of supplementation after the initial 9-week period of experimental feed without Cr supplementation. Two types of diet were applied: a standard-fat-and-fiber (S) diet and a high-fat–low-fiber (F) diet. Rats at 7 weeks of age were fed with an S diet or an F diet for either 9 weeks or 9 + 9 weeks. The following groups of rats were studied: Control S, the S diet plus the S diet; Control FS, the F diet and the S diet; Cr–Pic FS, the F diet and the Cr–Pic S diet; Cr–NPs FS, the F diet and the Cr–NPs S diet; Control FF, the F diet and the F diet; Cr–Pic FF, the F diet and the Cr–Pic F diet; Cr–NPs FF, the F diet and the Cr–NPs F diet. Values are means ± SD, n = 8, *p* ≤ 0.05 (two-way ANOVA/Tukey’s multiple comparisons test). In arteries from rats fed with the F diet for 9 weeks, both Cr–Pic and Cr–NPs increased relative O_2_^.−^ production. In arteries from rats fed with the F diet for 18 weeks, Cr–Pic but not Cr–NPs decreased elevated H_2_O_2_ production.

In arteries from rats fed the F diet for 9 weeks, relative O_2_^.−^ production did not change in the control FS group. However, both Cr–Pic (1.22-fold) and Cr–NPs (1.20-fold) increased the relative O_2_^.−^ production. This was not observed when the F diet was administered for 18 weeks (Figure 9A).

#### 3.4.4. Detection of Hydrogen Peroxide

In arteries from rats fed the F diet for 9 weeks, H_2_O_2_ production did not change in the control FS group (*p* = 0.5951). Neither Cr–Pic nor Cr–NPs modified that response. However, a significant increase (1.59-fold) was observed in the Cr–NPs group vs. the Cr–Pic group (Figure 9B).

In arteries from rats fed the F diet for 18 weeks, H_2_O_2_ production increased in the control FF group (1.96-fold). Cr–Pic decreased (0.73-fold) the elevated H_2_O_2_ production (Figure 9B). This was not observed for Cr–NPs (0.87-fold, *p* = 0.6087).

## 4. Discussion

We examined the cardiovascular effects of a pharmacologically relevant dose of 0.3 mg Cr/kg body weight [10] in the form of picolinate (Cr–Pic) and metal nanoparticles (Cr–NPs) in male Wistar Han rats administered in 9- to 16-week-old rats together with implementation of a high-fat–low-fiber (F) diet for either 9 or 18 weeks to investigate the role of dietary fat normalization.

It is well-known that a prolonged high-fat diet intake results in the development of chronic systemic inflammation, as well as tissue and organ dysfunction, and our study confirmed this effect in rats fed the F diet for 18 weeks.

In the functional studies, we demonstrated that Cr–Pic and Cr–NPs supplementation favor the COX-2 pathway (over 9 and 18 weeks of F-diet administration), which is also an attribute of a prolonged and regular F diet intake (in this study, it only occurred in 18 weeks, not 9 weeks, of F-diet administration). Surprisingly, Cr preparations induced more negative changes in arteries over 9 weeks of F-diet intake compared to 18 weeks, which resulted in (i) enhanced participation of NO derived from iNOS and (ii) an enhanced net effect of vasodilator prostanoids derived from COX-2 in vascular relaxation. However, it was Cr–NPs that induced an imbalance between the function of COX-2-derived vasodilator and vasoconstrictor prostanoids over 9 weeks rather than 18 weeks of feeding, in such a way that the net vasodilator effect of prostanoids other than PGI_2_ predominated in this group. A similar effect was seen in 18-week F-diet intake that had no added Cr (control FF group). We also noticed that both Cr–Pic and Cr–NPs increased the relative production of O_2_^.−^ in arteries from rats fed the F diet for 9 weeks; however, it was Cr–Pic but not Cr–NPs that decreased elevated H_2_O_2_ production in arteries from rats fed the F diet for 18 weeks.

This undesirable effect of Cr, especially related to Cr–NPs, was also observed in the blood and hearts of supplemented rats and was reflected in decreased blood plasma antioxidant capacity of water-soluble compounds and heart GSH+GSSG over 9 weeks of F-diet intake.

However, some beneficial effects of Cr–Pic and Cr–NPs were observed after 18 weeks of F-diet administration. In this study, an 18-week F diet decreased heart SOD and increased heart CAT, and both Cr–Pic and Cr–NPs modified that response to the level observed in control rats fed standard-fat-and-fiber diet. This suggests some beneficial effects that can be attributed to Cr; however, they are only seen when an F diet is consumed for a longer period (18 weeks instead of 9 weeks). Moreover, Cr–NPs decreased heart MDA levels in this group of rats, again pointing to some benefits of Cr–NPs, but only when an F diet is administered for 18 weeks.

It is worth mentioning that in our study, Cr preparation did not modify the blood pressure, the ACL in the blood, the vasodilator response to SNP, the contractile response to noradrenaline and high KCl, or the production of TXA_2_ and PGI_2_.

### 4.1. Vascular Effects of Experimental Feeding

Neither receptor-dependent nor independent mechanisms of contraction were involved in this study (research with noradrenaline and KCl). Moreover, dietary supplementation did not modify the relaxant response to SNP of endothelium-denuded rings, which is an endothelium-independent mechanism, indicating that the sensitivity of the smooth muscles to NO was not modified. This corresponds well with the fact that the blood pressure was also not modified. Comparable results were obtained by Abebe et al. [22], who reported no influence of Cr–Pic on vascular contraction and blood pressure; however, this study was conducted on spontaneously hypertensive rats (SHR) instead of normotensive Wistar Han rats. Nonetheless, Kopilas et al. [23] reported that Cr (III) reduced systolic blood pressure in mesenteric resistance arteries of sucrose-induced hypertension in SHR.

Next, we studied the endothelium-dependent relaxation of ACh. Our results showed that arteries from non-supplemented rats fed an F diet for 18 weeks (control FF group) responded with increased relaxation of ACh, and Cr–Pic decreased that response to a level corresponding with non-supplemented rats fed a standard-fat-and-fiber diet (control S group). This indicates that it was 18 weeks rather than 9 weeks of F-feeding that resulted in modified vascular relaxation, possibly through enhanced production of H_2_O_2_ and Cr–Pic. These functional results with Cr–Pic are in contrast with the results of Abebe et al. [22], who reported increased ACh-induced vasodilation in spontaneously hypertensive rats (SHR) supplemented with Cr–Pic. However, in these specific animal strains, vascular relaxation is already decreased due to the inherent hypertension.

As there was no change in response to NA, we could rule out the influence of vascular contraction on ACh-induced relaxation. Similarly, as the vasodilation to SNP was also not altered, we could rule out modification of the sensitivity of the smooth muscles to exogenous NO. Since NO is a major vasodilator agent in the rat aorta, which is a large elastic conduit artery, we concluded that the observed effect might be associated with increased NO production/release rather than modified sensitivity to NO.

Next, we observed that the specific iNOS inhibitor 1400 W reduced the vasodilation to ACh in Cr–Pic- and Cr–NPs-supplemented groups of rats fed an F diet for 9 weeks. Surprisingly, 1400 W did not modify this response over 18 weeks of F-diet intake. These results show that the sensitivity to NO derived from iNOS increased during these 9 weeks of feeding, and these same findings also point to a possible increase of NO production/release as a response to the proinflammatory mediators such as O_2_^.−^, whose production was elevated in our study. Since the sensitivity to NO derived from iNOS was not altered over 18 weeks of F-diet administration, there may be a possible compensatory effect when both Cr and an F diet are taken for prolonged periods; a conclusion that merits further study.

As there is an interplay between NO and COX-derived prostanoids in vascular tone regulation [6,14], our next step was to analyze the participation of COX derivates in that response. The non-selective COX inhibitor indomethacin had no effect on the vasodilatory response in control rats fed a standard-fat-and-fiber diet or an F diet for 9 weeks (control S and control FS group, respectively), which indicates that COX derivates do not participate in that response. Another possible explanation is that the sensitivity of the arteries to prostanoids was lost in these two controls.

Interestingly, vasodilation to both indomethacin and selective COX-2 inhibitor NS-398 was attenuated in preincubated arteries from Cr–Pic and Cr–NPs supplemented rats, which indicates that COX-2-derived prostanoids modulate the endothelium-dependent vasodilation in thoracic arteries of rats fed an F diet for 9 weeks. Since the same attenuation in vascular response was reported in control rats fed an F diet for 18 weeks (control FF) and Cr-supplemented FF rats, this only adds to our observation that raised COX-2 expression is the factor that characterizes the pro-inflammatory status and that Cr supplementation only enhances this process. An increased production of ROS further adds to this conclusion. It is worth mentioning that Cr–Pic further decreased the maximal vasodilator response to ACh during the 9 and 18 weeks of an F diet, but this was not observed with Cr–NPs. This can be partially explained by the fact that H_2_O_2_ production was not modified by Cr–NPs as it was by Cr–Pic (18 weeks of F diet).

Since both the non-selective COX inhibitor and the selective COX-2 inhibitor attenuated the ACh-induced response to the same degree, we hypothesized that increased participation of a strong vasodilator derived from COX-2 seems to play a key role in this response in Cr-supplemented rats. The available literature indicates that PGI_2_ is the most potent vasodilator in rat conduit arteries. However, TCP, which is a PGI_2_ synthesis inhibitor, did not modify that response in the two Cr-supplemented groups of rats fed an F diet for 18 weeks as it did for the control (control FF), which indicates that the sensitivity of these arteries to PGI_2_ had been lost. The same effect was observed in arteries from Cr–Pic-supplemented rats fed an F diet for 9 weeks. Surprisingly, Cr–NPs during the 9 weeks of feeding with an F diet increased sensitivity to PGI_2_, but this was not modified with TCP plus SQ-29,548 (a TP receptor inhibitor), possibly due to the increased H_2_O_2_ production also observed in aortas from affected controls fed a pro-inflammatory diet (an F diet) for 18 weeks.

Since other prostanoids modify vascular reactivity through TP receptor activation, pre-incubation with TP receptor antagonist SQ-29,548 was studied. However, SQ-29,548 did not modify that response in supplemented rats or the control rats, indicating that TP receptors did not participate in that response, which agrees with other reports [24,25].

Complex interactions have been reported between COX derivates, since the synthesis of one prostanoid is usually accompanied by changes in the synthesis of others, which can be shifted to the compensatory responses [26]. As there is crosstalk between the PGI_2_ and TXA_2_ systems, we analyzed vascular relaxation after the inhibition of PGI_2_ synthesis and TP receptors with TCP plus SQ-29,548. In the two control groups (standard-fat-and-fiber diet (control S) and F diet for 9 weeks (control FS)), pre-incubation with TCP plus SQ-29,548 reversed the decreased response to ACh caused by TCP, demonstrating a balance between PGI_2_ and TXA_2_ in these arteries. However, in arteries from rats fed the F diet for 18 weeks (control FF group), TCP plus SQ-29,548 did not modify the decreased ACh response caused by TCP. A similar effect to that described for the control FF group was also observed in Cr–NPs-supplemented rats fed a high-fat diet for 9 weeks, indicating the imbalance between these two prostanoids and the possible participation of prostanoids other than PGI_2_ and TXA_2_ through their specific receptors, rather than on TP receptors. This has already been confirmed in a functional study with NS-398, in which the vasodilator effect of prostanoid was decreased and neither TCP nor TCP plus SQ-29,548 reached the vasodilator level observed for NS-398. Surprisingly, in the other three experimental groups supplemented with Cr, sensitivity to endogenous PGI_2_ was lost (study with TCP and TCP plus SQ-29,548), which points to some imbalance between PGI_2_ and TXA_2_. This might be explained by the fact that the production of O_2_^.−^ and H_2_O_2_ was increased in the control arteries (control FF), while Cr supplementation decreased that production.

### 4.2. Cardiac Effects of Experimental Feeding

It is well-known that a high-fat diet modifies the metabolic pathway, leading to the generation of ROS that modulate the antioxidant defense mechanisms. Our results indicate that the hearts of rats fed an F diet for 18 weeks, contrary to 9 weeks, exhibited decreased SOD (an enzyme that degrades O_2_^.−^) and increased CAT (an enzyme that degrades H_2_O_2_), with a tendency to increase GSH+GSSG (ROS scavenger). The decreased blood-plasma antioxidant capacity of water-soluble compounds further supports our results. Surprisingly, the MDA level, which is a marker of lipid peroxidation, was not modified; this is in contrast with our previous results [5] and Noeman et al. [27]. Overall, our results have shown that a prolonged and regular F diet significantly modified the antioxidant defense mechanisms that are crucial for the development and progression of metabolic syndrome [28]. Irrespective of its form, supplementation with Cr–Pic and Cr–NPs increased SOD and decreased CAT in compromised rats over 18 weeks of a proinflammatory diet to the same level observed in rats fed with a standard-fat-and-fiber diet. Surprisingly, Cr–NPs decreased MDA; meanwhile, Cr–Pic exhibited a tendency to lower GSH+GSSG. This is in contrast with our previous results, which showed an increased level of MDA in the hearts of rats supplemented with Cr–Pic and Cr–NPs [5]. 

However, this prior experiment lasted for 9 weeks instead of 18 weeks; in our 9-week experiment, MDA did not change, and rats were fed with a different type of a high-fat diet. These results point to some beneficial effects of Cr preparations on the redox status, but only when an F diet is taken for a longer and regular period. Surprisingly, dietary intervention and reduction of F diet duration to 9 weeks completely modified the effects described above. At some point, 9 weeks of an F diet intake caused this, despite not modifying MDA, GSH+GSSG, SOD, CAT, or ACW. That is why neither MDA, SOD, nor CAT were changed by these two forms of Cr, as opposed to GSH+GSSG, which was reduced. However, it was Cr–NPs that further reduced antioxidant capacity in the blood. Our results agree with research conducted by Chen et al. [29], which suggested a positive effect of Cr on redox status in individuals that regularly consume a high-fat diet, and who are often obese. However, in healthy individuals within body mass guidelines, Cr preparations can function as pro-oxidant agents, initiating free-radical reactions that lead to oxidative stress [10].

## 5. Conclusions

Our study on rats confirmed the pro-inflammatory effect of an F diet administered for 18 weeks. Our results show that supplementation with Cr–Pic rather than with Cr–NPs is more beneficial in rats who regularly consume an F diet (18 weeks). On the contrary, in healthy organisms (9 weeks of an F followed by dietary fat normalization), both Cr–Pic and Cr–NPs can function as pro-oxidant agents, initiating free-radical reactions that lead to oxidative stress.

## Figures and Tables

**Table 1:** Composition of diets fed to rats (%) *.

Ingredient/Group	S	F
Casein	14.8	14.8
DL-methionine	0.2	0.2
Cellulose	8.0	3.0
Choline chloride	0.2	0.2
Cholesterol	0.3	0.3
Vitamin mix	1.0	1.0
Mineral mix	3.5	3.5
Maize starch	64	52
Rapeseed oil	8.0	8.0
Lard	-	17.0

* Chromium (III) picolinate (Cr–Pic) and chromium nanoparticles (Cr–NPs) were emulgated in dietary rapeseed oil, not in the mineral mixture (MX), and added into the diet.

**Table 2:** The influence of inducible nitric oxide synthase (iNOS) inhibitor (1400 W at 1 µM), the non-selective COX inhibitor (indomethacin at 10 µM), the selective cyclooxygenase-2 (COX-2) inhibitor (NS-398 at 10 µM), the thromboxane-A2 receptor (TP) antagonist (SQ-29,548 at 1 µM), the PGI2 synthesis inhibitor (tranylcypromine, TCP at 10 µM), or TCP plus SQ-29,548 on the vasorelaxant effects to acetylcholine of thoracic arteries of either chromium (Cr)-supplemented (9 weeks of supplementation) or not-supplemented (Control) rats. 0.3 mg Cr/kg of body weight as chromium picolinate (Cr–Pic) and chromium nanoparticles (Cr–NPs) were added after 9 weeks of experimental feed without Cr supplementation. Rats were fed a standard (S) diet for 18 weeks, and a high-fat–low-fiber (F) diet for either 9 weeks (FS group) or 18 weeks (FF group).

		Control Conditions *			1400 W			Indomethacin			NS-398			SQ-29,548			Tranylcypromine			TCP plus SQ-29,548		
		AUC	Emax (%)	pEC_50_	AUC	Emax (%)	pEC_50_	AUC	Emax (%)	pEC_50_	AUC	Emax (%)	pEC_50_	AUC	Emax (%)	pEC_50_	AUC	Emax (%)	pEC_50_	AUC	Emax (%)	pEC_50_
S	Control	305.3	96.16	7.967	308.0	96.38	7.950	287.2	92.37	7.921	254.9 *	90.84	7.675	259.6	88.87	7.743	257.9 *	−91.97	7.566 *	280.0	93.47	7.815
FS	Control	310.4	98.78	8.002	305.2	96.47	7.943	278.0 *	95.75	7.816	290.9	96.26	7.939	294.5	98.61	7.866	278.3 *	−97.01	7.632 *	311.3	97.54	7.996
	Cr–Pic	300.4	95.60	7.940	285.2 *	95.81	7.805	236.5 *	86.90 *	7.530 *	223.0 *	87.51 *	7.384 *	249.7	92.89	7.589	246.7	−91.48	7.597	247.5	89.71	7.536
	Cr–NPs	315.6	98.77	8.032	283.5 *	91.61	7.938	254.9 *	92.52 *	7.616 *	228.2 *	94.09	7.486 *	276.5	96.21	7.759	267.3 *	−94.70 *	7.641 *	253.8 *	92.49 *	7.571 *
FF	Control	331.5	97.63	8.238	343.9	96.77	8.285	298.6 *	95.93	7.867 *	261.2 *	94.13	7.562 *	301.5	97.05	7.904	276.9	−93.83	7.855	273.0 *	96.29	7.661 *
	Cr–Pic	302.3	98.81	7.943	278.3	94.88	7.710	233.9 *	86.18 *	7.605 *	203.1 *	83.53 *	7.471 *	296.5	97.64	7.858	294.9	−95.57	7.924	270.3	97.58	7.676
	Cr–NPs	311.7	95.31	8.124	295.4	93.71	7.977	288.2 *	95.10	7.805 *	232.4 *	86.34	7.503 *	285.3	95.27	7.826	305.5	−97.65	7.937	313.7	95.52	8.017

Values are based on the concentration–response curves. Data are expressed as means, *n* = 8, * *p* ≤ 0.05 compared with the control conditions, as determined by two-way ANOVA followed by Tukey’s post hoc test.

## Data Availability

Data supporting reported results are available on request.
